# Publication bias and the limited strength model of self-control: has the evidence for ego depletion been overestimated?

**DOI:** 10.3389/fpsyg.2014.00823

**Published:** 2014-07-30

**Authors:** Evan C. Carter, Michael E. McCullough

**Affiliations:** ^1^Department of Psychology, University of MiamiCoral Gables, FL, USA; ^2^Department of Ecology, Evolution and Behavior, University of MinnesotaSt. Paul, MN, USA

**Keywords:** self-control, self-regulation, ego depletion, publication bias, meta-analysis, small-study effects

## Abstract

Few models of self-control have generated as much scientific interest as has the limited strength model. One of the entailments of this model, the depletion effect, is the expectation that acts of self-control will be less effective when they follow prior acts of self-control. Results from a previous meta-analysis concluded that the depletion effect is robust and medium in magnitude (*d* = 0.62). However, when we applied methods for estimating and correcting for small-study effects (such as publication bias) to the data from this previous meta-analysis effort, we found very strong signals of publication bias, along with an indication that the depletion effect is actually no different from zero. We conclude that until greater certainty about the size of the depletion effect can be established, circumspection about the existence of this phenomenon is warranted, and that rather than elaborating on the model, research efforts should focus on establishing whether the basic effect exists. We argue that the evidence for the depletion effect is a useful case study for illustrating the dangers of small-study effects as well as some of the possible tools for mitigating their influence in psychological science.

## Introduction

For more than a decade, the proposition that self-control relies on a limited resource has been a mainstay of theorizing on self-control that has influenced researchers across psychology sub-disciplines, including social-personality (Inzlicht et al., 2006), clinical (Christiansen et al., 2012), health (Hagger, 2010), cognitive (Pohl et al., 2013), and consumer psychology (Baumeister et al., 2008). The limited strength model of self-control specifies that volitional acts (e.g., controlling impulses, overriding habitual responses, making choices) rely on a limited resource. Consequently, because stores of the requisite resource are depleted with use, attempts at self-control that follow previous acts of self-control will be less successful (a phenomenon called *ego depletion*; Baumeister et al., 2007; Bauer and Baumeister, 2011). To date, more than 200 experimental evaluations of the ego depletion hypothesis have been published.

The first empirical support for ego depletion was published in 1998 in two multi-experiment papers (Baumeister et al., 1998; Muraven et al., 1998), both of which have since become citation classics: a Google Scholar search conducted July 1st, 2014 returned 1172 citations for Muraven et al. (1998) and 2172 for Baumeister et al. (1998). The methods Muraven et al. (1998) and Baumeister et al. (1998) developed to test the limited strength model are straightforward: first, participants complete either a version of a task that is designed to be self-control-intensive (the “depletion” condition) or a version designed to require relatively little self-control (the control condition). Next, participants complete a different task—also designed to require self-control. We refer to this experimental method as *the sequential task paradigm*. If self-control relies on a limited resource, then participants in the depletion condition should perform worse on the second task than do those in the control condition. We refer to this pattern of results as *the depletion effect*.

Hagger et al. (2010) reported a meta-analysis of the 198 published tests of the depletion effect, concluding that the evidence was robust and replicable with an overall effect size of *d* = 0.62 [95% CI: (0.57, 0.67)]. Despite Hagger et al.'s (2010) conclusion, we show here that the seemingly strong support for the limited strength model is likely inflated by small-study effects (i.e., the tendency for studies with smaller samples to produce larger effect size estimates), perhaps to the extent that the ostensible evidence for the depletion effect may purely be an artifact of researchers' and editors' aversion to publishing null findings (i.e., publication bias, the most widely discussed small-study effect). We detail a promising method for detecting and correcting for small-study effects that is not currently in widespread use in Psychology, and based on our results, we argue that current efforts to refine the limited strength model (e.g., Inzlicht and Schmeichel, 2012; Kurzban et al., 2013) should be considered secondary in importance to determining whether truly convincing empirical support for the foundational finding of the model exists.

Although publication bias, as we will show, may be a problem for the published literature on the limited strength model, this problem is certainly not unique to the ego depletion literature. Indeed, publication bias very likely influences a disturbingly large proportion of research literatures in psychology (Bakker et al., 2012; Ferguson and Brannick, 2012). Our focus on the limited strength model was born out of our own difficulties in producing the depletion effect, and we believe that a closer look at Hagger et al.'s (2010) meta-analysis can function as a case study on the issue of small-study effects that will be of great use to researchers in psychology who are interested in either the topics of self-control or meta-analysis.

Like many others, we have found the limited strength model to be a helpful tool for developing theory (e.g., McCullough and Willoughby, 2009; McCullough and Carter, 2011). However, despite our initial confidence in the evidence for the model, our attempts at using the sequential task paradigm to generate novel experimental work were met with failure (Carter and McCullough, 2013a). In response to our inability to produce the basic depletion effect in our own work, we turned to Hagger et al.'s (2010) meta-analysis for guidance on how to design a successive experiment so as to maximize our likelihood of success in the future (e.g., by determining which types of self-control tasks tend to produce the largest depletion effect and the sample sizes needed to achieve acceptable statistical power). However, upon reading Hagger et al. (2010), we realized that their efforts to estimate and account for the possible influence of publication bias and other small-study effects had been less than ideal, given the methods available at the time of its publication. In other words, we were concerned that Hagger et al.'s (2010) estimates of the depletion effect might have been inflated to a completely unknown degree, and as a result, represented a less useful synthesis of the literature on the depletion effect (i.e., one that could be used to guide future research) than would have otherwise been the case.

Importantly, the possibility that Hagger et al.'s (2010) methods for estimating and correcting for small-study effects were less than ideal does not, in and of itself, invalidate their conclusions, but given the influence and popularity of the limited strength model, we felt that there was a need for an estimate of the depletion effect that was maximally useful for guiding future research (i.e., an estimate of the depletion effect that was relatively robust to small-study effects). Therefore, our first goal here was to more thoroughly evaluate the evidence for small-study effects (particularly publication bias) in Hagger et al.'s (2010) dataset using recently developed methods and to re-estimate the magnitude of the depletion effect while taking into account any evidence for small-study effects. To do so, we applied a set of three statistical techniques to Hagger et al.'s (2010) published dataset: (1) the binomial test described by Ioannidis and Trikalinos (2007a); (2) the trim and fill method (Duval and Tweedie, 2000a,b); and (3) an extension of Egger's regression test (Egger et al., 1997; Stanley, 2008; Moreno et al., 2009a). The first method identifies signs of publication bias, whereas the latter two identify and correct for its influence.

The application of the extension of Egger's regression test and the binomial test to Hagger et al.'s full dataset have been reported previously (Carter and McCullough, 2013b). The current paper details our follow-up to that initial statistical inquiry, which clearly suggested that the Hagger et al.'s (2010) estimate of the depletion effect was heavily biased. The follow-up analyses presented here include (1) the application and discussion of the popular trim and fill method, (2) the construction of contour-enhanced funnel plots, and (3) a subset analysis designed to address the possibility of alternative explanations to our results—namely, that something other than publication bias has caused the small-study effects we observed. These additions provide a much fuller picture of the data, as well as support for our initial conclusion that the evidence for the depletion effect has likely been severely overestimated due to publication bias. It is worth noting that Hagger and Chatzisarantis (2014) have commented on our previous work (Carter and McCullough, 2013b), and have independently reproduced those results.

A second, concurrent goal of this paper was to highlight the use of the extension of Egger's regression test as a means of obtaining an estimate of the true underlying effect that is corrected for publication bias (Moreno et al., 2009a; Stanley and Doucouliagos, 2013). This promising approach, and other related regression-based methods, are currently in use in the fields of economics (e.g., Stanley, 2005, 2008; Doucouliagos and Stanley, 2009; Havranek, 2010; Costa-Font et al., 2011) and medicine (e.g., Moreno et al., 2009a,b; Hemingway et al., 2010; Nüesch et al., 2010; Rücker et al., 2011a,b), but (to our knowledge) has seen only a single use in Psychology (specifically, in the area of judgment and decision making research; Renkewitz et al., 2011). These regression-based methods for producing a meta-analytic estimate of the true underlying effect that is robust to publication bias are intuitive and easy to use, and the current paper provides readers with the information needed to begin using the regression-based methods that we discuss (see the Appendix in the Supplementary Materials). Furthermore, we also provide the necessary discussion of critical issues that should be considered when undertaking this type of analysis—that is, statistical heterogeneity and the difference between small-study effects and publication bias.

It is well known that the accuracy of meta-analysis depends utterly on the representativeness of the sample of studies that are analyzed. One way in which a sample can become unrepresentative is through publication bias (Begg and Berlin, 1988; Rothstein et al., 2005; Sutton, 2009; Bakker et al., 2012; Ferguson and Heene, 2012; Francis, 2012a), that is, when the likelihood of a study appearing in that sample is influenced by the study's results. For example, publication status is influenced by the presence of significant results, and published studies are more visible and accessible to researchers performing meta-analyses (Sutton, 2009). Therefore, studies with significant, theory-supporting results tend to be more likely to end up in meta-analytic samples. The problem is that if a sample of studies is biased, in that findings that support the existence of a particular effect are over-represented, the magnitude of the meta-analytic estimate of the effect will be overestimated (e.g., Stanley, 2008; Sutton, 2009; Bakker et al., 2012; Francis, 2012a).

Encouragingly, psychological researchers seem to be becoming increasingly aware of the danger of publication bias (e.g., LeBel and Peters, 2011; Bakker et al., 2012; Ferguson and Heene, 2012; Francis, 2012a,b; Schimmack, 2012). In particular, one method for detecting signs of publication bias by examining the statistical power of a set of studies (proposed by Ioannidis and Trikalinos, 2007a) has recently gained popularity in psychology (Francis, 2012a,b; Schimmack, 2012; cf. Simonsohn, 2012). This power-based method is founded on the idea that one can use the average statistical power of a set of studies as the likelihood that any single study in the set would reach statistical significance. Under that assumption, a binomial test can be used to examine whether the observed number of significant findings exceeds the number that would be expected given the average power of the entire set. Smaller *p*-values for this test indicate an excess of significant results, which suggests that null findings are underrepresented in the set, possibly due to publication bias. The average power for a set of studies can be calculated based on a meta-analytic estimate of the effect size, but it can also be calculated based on the effect size estimate given in each study (Schimmack, 2012). A recent application of this method to 44 eligible papers published in one of the most prestigious journals (at least for psychologists who run experiments) between 2009 and 2012 revealed that 82% of those papers evinced fewer statistically non-significant results than would have been expected by chance, implying that publication bias might be extraordinarily common among the papers in this particular journal (Francis, 2014).

In their review of meta-analyses published in top psychological journals between 2004 and 2009, Ferguson and Brannick (2012) reported that researchers in psychology have historically favored two other methods for approaching the problem of publication bias. The two most commonly used statistical methods designed specifically to deal with publication bias were the failsafe N (used in 22% of meta-analyses in Ferguson and Brannick's, 2012 sample) and the trim and fill (used in 24% of Ferguson and Brannick's, 2012 sample). Importantly, the failsafe N, of which there are several variations, is based on unrealistic assumptions, and has been widely criticized (e.g., Becker, 2005; Ioannidis, 2008; Sutton, 2009; Ferguson and Heene, 2012). Essentially, this method is designed to provide meta-analysts with license to ignore the issue of publication bias by estimating the number of studies with an average effect size of zero that would have to exist outside of the meta-analytic sample to bring the meta-analytic estimate to zero if they were included. If this number is sufficiently large, then the effect in question is considered to likely not be due entirely to publication bias. Though conceptually attractive, the failsafe N has been referred to as “nothing more than a crude guide” (Sutton, 2009, p. 442), and its use is thought to have possibly led to complacency about publication bias (Sutton, 2009). It is fair to say that its use is generally not recommended (e.g., Becker, 2005; Ioannidis, 2008). For our current purposes, it is important to note that Hagger et al.'s (2010) use of a version of the failsafe N (Rosenberg, 2005) revealed that 50,445 unpublished experiments on the depletion effect with an average effect of zero would have to exist outside of their dataset to bring their estimate of *d* = 0.62 down to zero. Based on this number, they concluded that “it was highly unlikely that sufficient studies with null effects would exist to reduce the ego-depletion effect to a trivial value” (Hagger et al., 2010, p. 508).

The trim and fill (Duval and Tweedie, 2000a,b), the other method commonly used in psychological research (Ferguson and Brannick, 2012), represents a considerable improvement over the failsafe N. This method is based on how the relationship between effect size estimates and their standard errors in a set of studies changes in the presence of publication bias (see Bakker et al., 2012 for a review of this topic). To understand this process, imagine a situation in which the effect in question is truly zero. Estimates of this effect from individual studies will vary around zero due to sampling error, providing both under- and overestimates of the true effect. Therefore, when a set of studies measuring this effect are plotted with the standard errors of effect size estimates on the vertical axis and the magnitude of those effect sizes on the horizontal axis (a so-called funnel plot; Light and Pillemer, 1984), individual studies should scatter into a symmetrical funnel shape. The funnel shape results from the fact that the estimates with the highest precision (i.e., those with the smallest standard errors, and thus, the smallest confidence intervals) will cluster tightly around zero (the true effect), and less precise estimates will spread evenly and randomly to the right and left of zero as standard errors increase. Imagine further that, for this hypothetical null effect, there is a strong belief among researchers that the effect is positive and non-zero, so that both significant underestimates of the effect (i.e., the left side of the funnel) and accurate non-significant estimates of the effect (i.e., the center of the funnel around zero) are less likely to get published due to publication bias. If publication status influenced the likelihood of individual studies being included in the funnel plot in this manner, then the result would be a distortion of the funnel plot's symmetry because of the paucity of unpublished studies (which would tend to disproportionately make up the points around and to the left of zero). Due to the asymmetry in the funnel plot, a positive correlation between the magnitude of the effect sizes and their standard errors would emerge because more precise studies (i.e., those with smaller standard errors) will have estimates that are closer to zero (i.e., the true effect size).

The trim and fill estimates the number of missing studies in a dataset by “trimming” the funnel plot until it is symmetrical and then “filling” in both sides of the funnel in a way that maintains symmetry. Following the imputation (“filling in”) of the missing effect sizes, the underlying effect size is re-estimated using standard meta-analytic methods. In simulation studies, the trim and fill has been shown to reduce the bias introduced into meta-analysis via publication bias; however, it apparently also tends to under-correct for publication bias, produce incorrect confidence intervals, and occasionally generate false positives (Terrin et al., 2003; Peters et al., 2007; Moreno et al., 2009a). Based on these findings, some methodologists do not recommend its use (Moreno et al., 2009a).

Like the trim and fill, Egger's regression test is based on the funnel plot (Egger et al., 1997; Egger and Sterne, 2005); however, unlike the trim and fill, this test does not assume that publication bias has led to funnel plot asymmetry. Instead, this method quantifies the relationship between effect size estimates and their standard errors, regardless of whether that relationship was produced by publication bias or some other small-study effect. One way that Egger's regression can be described is as a weighted least squares (WLS) regression model in which effect size is predicted by standard error (weighted by the inverse of standard error squared; see the Appendix in the Supplementary Materials). The significance of the coefficient associated with standard error (i.e., the slope coefficient, *b*_1_) in the regression model is interpreted as a test of funnel plot asymmetry. Historically, only the slope coefficient of Egger's regression was interpreted; however, the use of this model has been expanded, apparently first by Stanley (2005), who interpreted the model's intercept (*b*_0_) as an estimate of the underlying effect size when standard error = 0. In other words, one can extrapolate from the regression line to an estimate of the effect size for a hypothetical, perfectly precise study (Stanley, 2005). Therefore, via a weighted least squares (WLS) regression model, one can both assess funnel plot asymmetry (the original Egger's regression test) and estimate an overall effect that is theoretically uninfluenced by publication bias. This model is sometimes referred to as a “precision-effect test” (PET; Stanley, 2005).

Other regression-based methods have also been proposed (for a review, see Moreno et al., 2009a). For example, simulation studies revealed that, although highly accurate when the true underlying effect was zero, PET tended to over-correct for publication bias, underestimating the true effect when it was non-zero (Stanley and Doucouliagos, 2007). In response to this result, Stanley and Doucouliagos (2007) proposed that the variance (i.e., standard error squared) be used as a predictor instead of standard error (a method that is sometimes referred to as a “precision-effect estimate with standard error,” or PEESE). Because PET is more accurate when the true underlying effect is zero, and PEESE is more accurate when the true effect is non-zero, Stanley and Doucouliagos (2013) further proposed the use of a conditional estimator (referred to as PET-PEESE): if one can reject the null hypothesis that *b*_0_ = 0 using PET, then *b*_0_ from PEESE should be used as the best estimate of the true effect. However, if one cannot reject the null hypothesis that *b*_0_ = 0 using PET, then *b*_0_ from PET should be used as the best estimate [see Stanley and Doucouliagos (2013) for a full treatment of the logic behind the conditional nature of PET-PEESE]. Simulation studies show that regression-based methods provide highly accurate estimates of underlying effects (Stanley and Doucouliagos, 2007, 2013; Stanley, 2008; Moreno et al., 2009a; Stanley et al., 2010; Rücker et al., 2011a,b). Furthermore, these approaches outperform other methods, particularly the trim and fill, which, as mentioned above, tends to under-correct for publication bias (Moreno et al., 2009a; Rücker et al., 2011a).

In addition to the numerous favorable simulation studies, there exists one particularly impressive application of regression-based methods to correct for the influence of publication bias on a meta-analytic estimate. Turner et al. (2008) published a report in which they compared the results from the 74 phase II and phase III trials of antidepressants registered with the US Food and Drug Administration (FDA) to the results of the set of 50 of those same studies that were eventually published in peer-reviewed journals. Whether a given study was eventually published was related to the outcome of the study (i.e., publication bias): of those trials whose results were eventually published, 94% yielded positive results, whereas only 51% of the full set of FDA-registered trials yielded positive results. Turner et al. (2008) reported that the meta-analytic estimate of the underlying effect (given as Hedges's *g*, an unbiased form of Cohen's *d*) derived from the set of FDA-registered trials was *g* = 0.31. Turner et al. (2008) also meta-analyzed only the set of those studies that had been published and found that the estimate of the underlying effect was larger, *g* = 0.41. When PET-PEESE was applied to the published dataset, *b*_0_ = 0.19 from PET, but the null hypothesis that *b*_0_ = 0 was rejected (i.e., there was evidence of a true underlying effect). Therefore, *b*_0_ from PEESE was calculated, as previous simulations had shown that it would provide the most accurate estimate of the underlying effect (Stanley and Doucouliagos, 2007, 2013; Moreno et al., 2009a). In this case, *b*_0_ from PEESE was *b*_0_ = 0.29. Obviously, this latter estimate of the effect size is only trivially different from the estimate of *g* = 0.31 that resulted from the meta-analysis of the full, unbiased set of studies (Moreno et al., 2009b; Stanley and Doucouliagos, 2013). Thus, the use of PET-PEESE appears to be an excellent option for approximating an unbiased effect in the face of publication bias. Importantly, both PET and PEESE can be run using any statistical software that supports regression (see the Appendix in the Supplementary Materials).

Two clarifications regarding the validity of regression-based methods are necessary. First, as mentioned, regression-based methods do not specifically assume that publication bias has caused funnel plot asymmetry, but rather, model “small-study effects” (Rücker et al., 2011a,b). The term small-study effects refers to a collection of (usually unknown) influences that cause smaller studies to provide systematically different effect size estimates than those provided by larger studies. For example, if some dependent measures require more resources to collect *and* yield larger effect size estimates, then funnel plot asymmetry will result because studies using these measures will have both smaller sample sizes and larger effect size estimates than studies that do not use these measures. Publication bias is another example of a small-study effect, since, as described above, it results in smaller studies providing larger estimates of the underlying effect than larger studies. It is critical to keep this point in mind when interpreting the results of methods based on funnel plot asymmetry: a correlation between effect size and sample size (or standard error) is not necessarily due to publication bias, and it is the meta-analyst's responsibility to explore other possible explanations (Moreno et al., 2009a; Rücker et al., 2011a).

Although caution is clearly necessary when interpreting coefficients from regression-based methods, one should not make the mistake of thinking that the coefficients provided by these models are meaningless outside of clear-cut cases of publication bias. Depending on the type of small-study effects, the most useful information provided by a meta-analysis might be an estimate of the true underlying effect that statistically controls for the influence of small-study effects (publication bias or otherwise), and this is exactly what regression-based methods provide. Note that the same cannot be said for the trim and fill, since the trim and fill corrects the estimate of the underlying effect by imputing studies that would be missing in the presence of publication bias, and then adding them to the existing meta-analytic sample—that is, the logic underlying the trim and fill is specific to publication bias, whereas the logic underlying regression-based methods is more general. Thus, at least in principle, the corrected estimate provided by the trim and fill is less useful than that provided by regression-based methods.

The second important clarification about the validity of regression-based methods regards their performance in the presence of between-study heterogeneity (i.e., variation in the effect size estimates provided by the individual studies). There are multiple forms of between-study heterogeneity, but the most important one for the present discussion is statistical heterogeneity (Higgins and Thompson, 2002). Statistical heterogeneity is defined as variation between effect size estimates from individual studies in a meta-analytic sample that is due to some source other than random sampling variance—that is, statistical heterogeneity implies systematic, meaningful differences in individual estimates of underlying effects. Estimates of statistical heterogeneity in a meta-analytic sample may indicate that the studies in the sample are not measuring the same underlying effect in the population, and use of the methods described above in the presence of statistical heterogeneity is controversial (e.g., Ioannidis and Trikalinos, 2007b; Ioannidis, 2008; Sterne et al., 2011). However, it is important to realize that small-study effects, including publication bias, may be the cause of statistical heterogeneity in some samples (Rücker et al., 2011a), so it is not appropriate to recommend that these methods never be applied to meta-analytic samples that show signs of statistical heterogeneity. Nonetheless, simulation studies have routinely shown that the performance of the trim and fill suffers in the presence of moderate to large amounts of statistical heterogeneity (Terrin et al., 2003; Peters et al., 2007; Moreno et al., 2009a), and that performance of regression-based methods suffers as well, albeit less so (e.g., Moreno et al., 2009a; Stanley and Doucouliagos, 2013). In the face of large amounts of statistical heterogeneity [e.g., an *I*^2^ statistic of 50% or more, which indicates that half or more of the observed between-study variability is due to sources other than sampling error (Higgins and Thompson, 2002)], it has been recommended that inference from methods based on funnel plot asymmetry be undertaken with caution (Sterne et al., 2011). Additionally, it has also been recommended that these methods not be applied to meta-analytic samples that include fewer than 10 studies (Sterne et al., 2011).

The regression-based methods discussed above, although promising, have not yet gained popularity in psychological science. As mentioned above, one of our goals here was to highlight the advantages of regression-based methods for assessing and correcting for small-study effects, in the hopes of encouraging researchers in psychology to begin applying and studying these techniques. A simultaneous goal was to provide a case study of the application of this technique, as well as other popular methods, that would be of interest to large number of researchers. We believe that Hagger et al.'s (2010) work on the depletion effect is a good candidate for such a case study for two reasons. First, Hagger et al.'s (2010) meta-analysis seems to exemplify the types of meta-analyses that are currently conducted in the psychological literature (Ferguson and Brannick, 2012). Second, the undeniable popularity of the limited strength model marks Hagger et al.'s (2010) dataset as particularly useful to a large number of researchers, and an accurate estimate of the depletion effect that is robust to small-study effects would likely be of great interest to researchers attempting to design future experiments (e.g., enabling them to estimate the effect size they should assume when calculating target sample sizes so that adequate statistical power is realized). To this end, we have applied PET-PEESE, along with the trim and fill and the binomial test, to Hagger et al.'s (2010) previously published meta-analysis on the depletion effect. We also demonstrate one possible way of handling significant statistical heterogeneity and the ruling out of possible small-study effects beyond publication bias.

## Materials and methods

All analyses were conducted using R (R Development Core Team, 2011); see the data sheet in the Supplementary Materials for data and scripts. Data were obtained from Martin Hagger, and further uses of these data should be acknowledged as such (Hagger et al., 2010).

### Primary analyses

Martin Hagger kindly provided us with the coded effect sizes^1^ for each experiment and the *n*s for the depletion and control groups. First, we re-estimated the standard fixed-effect and random-effects meta-analysis models. Second, the meta-analytic sample was evaluated for an excess of statistically significant findings (Ioannidis and Trikalinos, 2007a): we conducted the binomial test with power calculations based on both the fixed-effect and random-effects estimates of the underlying effect, as well as the estimates of the depletion effect provided by the individual experiments. Third, we applied the trim and fill method (to both the fixed-effect and random-effects models) and PET-PEESE.

#### Secondary analyses: addressing heterogeneity and alternative explanations

Hagger et al. (2010) explored significant statistical heterogeneity in their dataset by investigating whether the overall depletion effect varied by several experiment characteristics (e.g., the types of tasks used to induce or measure the depletion effect). To do so, Hagger et al. (2010) divided their dataset into subsamples of experiments that shared these characteristics and meta-analyzed the resulting subsamples separately. The results from this analysis suggested that few experiment characteristics moderated Hagger et al.'s (2010) estimate of the depletion effect (i.e., the magnitude of the effect changed little across subsamples). We used the subsamples created by Hagger et al.'s (2010) examination of moderating influences as a means of addressing the issues of statistical heterogeneity and small-study effects: first, because significant statistical heterogeneity in a meta-analytic sample can hamper the methods we used, as discussed above, it was prudent to apply them to more homogeneous subsamples. Second, funnel plot asymmetry can result from publication bias, but also from other small-study effects, so applying our methods to subsets of Hagger et al.'s (2010) dataset that possibly account for any positive correlation between standard error and effect size would help us to rule out possible alternative explanations. In other words, if small-study effects were the result of a particular experiment characteristic, rather than publication bias, analyzing the subsamples associated with that characteristic should reveal the source of funnel plot asymmetry. Therefore, we examined the sets of subsamples created by Hagger et al.'s (2010) moderator analyses for experiment characteristics that appeared to both (1) account for statistical heterogeneity and (2) create correlations between sample size and effect size (i.e., represent potential small-study effects) across experiments.

For two reasons, the best candidate moderator variable seemed to be Hagger et al.'s (2010) categorization of experiments by the “sphere of self-control” tapped by the second task. First, three of the four subsamples created by dividing up the total sample on the basis of this categorization scheme showed non-significant statistical heterogeneity. Second, one subsample, the “choice and volition” subsample, had the highest average sample size (*n* = 162.5 per experiment) and the lowest meta-analytic effect size estimate (*d* = 0.22) of all subsamples created in the moderator analysis—that is, the sphere of self-control tapped by methods used in individual experiments may have represented a study-specific characteristic that systematically created a positive correlation between standard error and effect size that could be mistaken as evidence for publication bias. Thus, we anticipated that applying our analyses to these subsamples would allow us to derive estimates from samples devoid of statistical heterogeneity, as well as to examine a possible alternative explanation to publication bias—specifically, that funnel plot asymmetry was caused by the fact that the choice and volition subsample produced both larger samples and smaller effect size estimates. Spheres were defined by Hagger et al. (2010) as “Controlling impulses” (e.g., tasks that required participants to resist temptation or override habits; *k* = 104), “Cognitive processing” (e.g., tasks that required the maintenance of a high level of cognitive effort; *k* = 47), “Choice and volition” (tasks that required participants to actively make choices; *k* = 8), and “Social processing” (tasks that required participants to respond appropriately in social contexts; *k* = 33). We applied the methods described above to each of these four subsamples^2^.

Finally, we created contour-enhanced funnel plots (Peters et al., 2008) for the total sample and the four subsamples. Contour-enhanced funnel plots are funnel plots in which the area of statistical non-significance is highlighted. When funnel plot asymmetry is due to studies missing primarily from the area of non-significance, one's confidence that asymmetry is due to publication bias, rather than other small-study effects, should increase (Peters et al., 2008).

## Results

**Figure 1:**
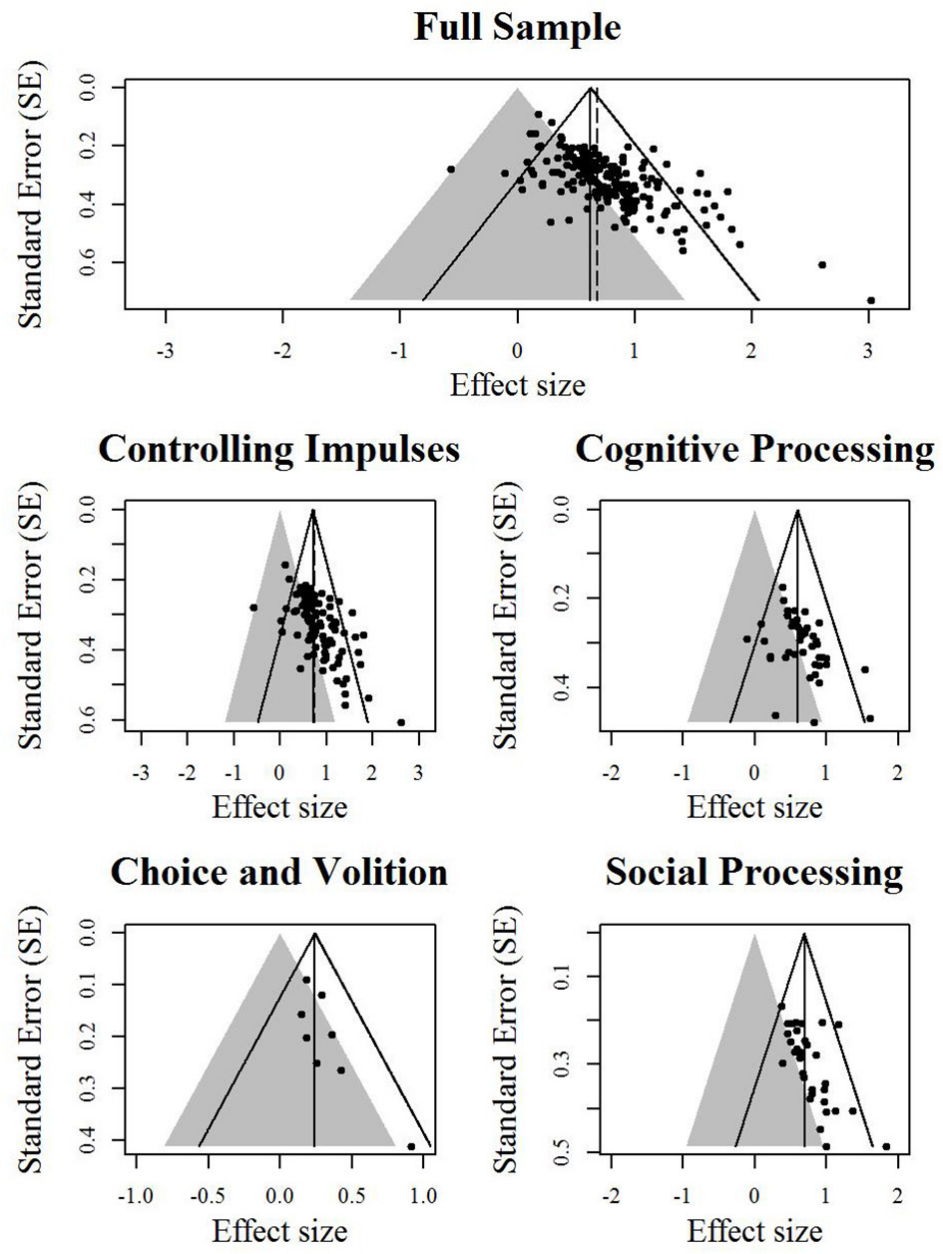
**Contour-enhanced funnel plots**. Effect sizes in the gray area are statistically non-significant. The solid angled lines represent the bounds within which 95% of studies should fall if there is no statistical heterogeneity. The solid vertical line represents the estimate for the overall effect from the fixed-effect model. The dashed vertical line represents the estimate of the overall effect from the random-effects model.

The fixed-effect and random-effects meta-analysis models (Table 1), the two versions of the binomial test (Table 1), the trim and fill (Table 2), and PET-PEESE (Table 3) were applied to the overall sample and to each subsample of effect sizes. (Note that the results for the binomial test and PET-PEESE applied to the full sample—in which outliers were modified, per Hagger et al. (2010)—appear in Carter and McCullough (2013b). All other analyses reported here, including all analyses involving the four “spheres of self-control” subsamples, are unique to this article). Contour-enhanced funnel plots (also unique to this article) are displayed in Figure 1. As recommended, the binomial test was conducted as a one-tailed test (Ioannidis and Trikalinos, 2007a), and *p* < 0.10 was used as the cutoff for tests of funnel plot asymmetry (Egger et al., 1997).

**Table 1:** **Standard meta-analysis models and *p*-values for the binomial tests**.

**Sample**	**Standard meta-analysis models**			**Statistical power and *p*-values for the binomial tests**			
	**FE: Overall effect**	**RE: Overall effect**	**Heterogeneity: *Q* and *I*^2^**	***k* with *p* < 0.05**	**Pow_ind_(*p*)**	**Pow_FE_ (*p*)**	**Pow_RE_ (*p*)**
Full	0.62 (0.58, 0.66)	0.68 (0.63, 0.74)	320.68; 38.6%	151	0.63 (5.63e-05)	0.55 (3.72e-10)	0.62 (8.32e-06)
CI	0.71 (0.65, 0.77)	0.75 (0.67, 0.83)	167.83; 38.6%	79	0.65 (0.012)	0.62 (0.001)	0.66 (0.015)
CP	0.60 (0.52, 0.68)	NA	43.03; 0%	34	0.58 (0.032)	0.54 (0.009)	NA
CV	0.24 (0.13, 0.35)	NA	4.52; 0%	3	0.38 (0.65)	0.31 (0.47)	NA
SP	0.69 (0.60, 0.78)	NA	29.11; 0%	32	0.69 (8.19e-05)	0.67 (2.53e-05)	NA

*Full = the full sample; CI = controlling impulses subsample; CP = cognitive processing subsample; CV = choice and volition subsample; SP = social processing subsample; FE = fixed-effect; RE = Random-effects. All p-values for overall effects and for the Q statistics, with the exception of for the CP, CV, and SP subsamples, were less than 0.001. Numbers given in parentheses are the lower and upper limits of the 95% confidence intervals. For each binomial test, average power was calculated from three possible sources: Pow_ind_ = power based on the effect sizes reported within individual experiments; Pow_FE_ = power based on the fixed-effect meta-analysis estimate of the overall effect size; Pow_RE_ = power based on the random-effects meta-analysis estimate of the overall effect size*.

**Table 2:** **Results from the trim and fill**.

**Sample**	**Trim and fill**		
	**“Filled” studies**	**FE: Overall effect**	**RE: Overall effect**
Full	73	0.48 (0.44, 0.51)	0.50 (0.44, 0.56)
CI	36	0.55 (0.50, 0.60)	0.56 (0.48, 0.65)
CP	12	0.51 (0.44, 0.56)	0.51 (0.43, 0.60)
CV	2	0.22 (0.11, 0.33)	0.22 (0.11, 0.33)
SP	13	0.56 (0.48, 0.64)	0.57 (0.46, 0.67)

^***^p < 0.001; ^**^p < 0.01; ^*^p < 0.05; ^†^p < 0.10. Full = the full sample; CI = controlling impulses subsample; CP = cognitive processing subsample; CV = choice and volition subsample; SP = social processing subsample; FE = fixed-effect; RE = Random-effects. All overall effects are significant, p < 0.001. Numbers given in parentheses are the lower and upper limits of the 95% confidence intervals.

**Table 3:** **Results from PET-PEESE**.

**Sample**	**PET**		**PEESE**	
	*****b***_0_**	*****b***_1_**	*****b***_0_**	*****b***_1_**
Full	−0.10 (−0.23, 0.02)	2.72^***^	0.25 (0.18, 0.32)	4.74^***^
CI	−0.24 (−0.50, 0.02)	3.19^***^	0.26 (0.13, 0.40)	4.76^***^
CP	0.02 (−0.35, 0.39)	2.08^†^	0.33 (0.14, 0.51)	3.37^**^
CV	0.06 (−0.14, 0.27)	1.25^**^	0.16 (0.06, 0.26)	3.48^*^
SP	0.18 (−0.10, 0.47)	1.94^***^	0.45 (0.30, 0.60)	3.32^***^

*** p < 0.001;

** p < 0.01;

* p < 0.05;

† *p < 0.10. Full = the full sample; CI = controlling impulses subsample; CP = cognitive processing subsample; CV = choice and volition subsample; SP = social processing subsample. For PET and PEESE, b_0_ = the intercept (i.e., the corrected estimate of the overall effect), b_1_ = the coefficient for standard error or variance (i.e., the test for funnel plot asymmetry). Numbers given in parentheses are the lower and upper limits of the 95% confidence intervals*.

First, in all but the choice and volition subsample, binomial tests indicated that the observed number of significant findings exceeded the expected number (Table 1). Moreover, it is clear that average power is lower than the recommended 0.80 in all cases, ranging from 0.31 to 0.69.

Second, the trim and fill method required that each sample be increased by 25–39% to achieve funnel plot symmetry (Table 2), and in all but the choice and volition subsample, the estimate of the overall effect was reduced by 15–26% as a result of the trim and fill procedure. Examination of the contour-enhanced funnel plots (Figure 1) suggests that asymmetry is mainly due to a lack of data points in the area of statistical non-significance (except for the choice and volition subsample).

Third, according to the coefficients in the regression models, all samples showed clear evidence for funnel plot asymmetry. Most notably, results from applying PET-PEESE clearly suggest that the true underlying effect for the overall sample and each of the subsamples is not distinguishable from zero (Table 3): in each case, *b*_0_ was not statistically significant for PET, suggesting that *b*_0_ from PEESE will be an overestimate of the true effect and that the least biased estimate is given by *b*_0_ from the PET models [the mathematical explanation for this conditional approach is described by Stanley and Doucouliagos (2013), and their accompanying simulation experiments further support its use]. Note that results from PET-PEESE for the choice and volition subsample should be treated with caution, however, since it is made up of fewer than 10 experiments (Sterne et al., 2011).

## Discussion

Our findings suggest that the published literature on the depletion effect is clearly influenced by small-study effects, and as a result, overestimates the strength of the phenomenon. Furthermore, it would appear that this overestimation is likely due to publication bias, rather than some other small-study effect: the results from the binomial tests and visual inspection of the contour-enhanced funnel plots suggests that asymmetry is due to a conspicuous lack of statistically non-significant findings, and our subsample analysis suggests that controlling for the most likely source of small-study effects (i.e., between-study differences in the methods used to measure dependent variables) does not eliminate funnel plot asymmetry. The application of a regression-based method (i.e., PET-PEESE), which was designed to correct for small-study effects, including publication bias, and which current evidence suggests provides the most unbiased estimate of the true underlying effect (e.g., Stanley, 2008; Moreno et al., 2009a; Rücker et al., 2011a; Stanley and Doucouliagos, 2013), indicates that the depletion effect is not distinguishable from zero. Put succinctly—and counter to our own personal intuitions about how human psychology works—after controlling for the influence of small-study effects, our results do not support the claim that the depletion effect is meaningfully different from zero.

One limitation to our analysis is that we addressed the potential overestimation of the depletion effect purely via statistical techniques rather than by trying to incorporate relevant unpublished results. Although important, this limitation must be qualified by two points. First, although conscientious efforts at retrieval of unpublished work are worthwhile, such efforts alone do not obviate concern about overestimation due to publication bias because there are serious barriers to collecting the results of unpublished studies. For example, many null findings are not only unpublished, but also lack any form of written documentation, and thus, are very difficult to track down. One apparent consequence of these barriers is that the successful collection of a sample of unpublished studies that is unbiased (i.e., a sample that is representative of all unpublished studies) is quite rare, so the inclusion of unpublished studies may introduce additional unknown forms of bias (Ferguson and Brannick, 2012).

Second, it may actually be impossible to collect certain null findings because these findings have been transformed into positive findings through statistical adjustments or the exercise of undisclosed “researcher degrees of freedom” (Simmons et al., 2011) that inflate the chances that results will reach statistical significance. Examples of such practices include excluding outliers *post-hoc*, using multiple outcome measures and only reporting results for ones that reached statistical significance, and optional stopping—that is, halting data collection to test for significance and resuming data collection if significance is not found. A recent survey of researchers in psychology suggests that these practices are frequent—for excluding data *post-hoc*, failing to report all dependent measures, and optional stopping, the estimated prevalence was 62%, 78%, and 72%, respectively—so the bias they introduce is a concern for all literatures in psychology (John et al., 2012). If the exaggeration of evidence for an effect is due to the use of undisclosed researcher degrees of freedom, even a complete collection of unpublished results cannot obviate this influence because *null results will have been turned into statistically significant results* (Ferguson and Heene, 2012). To be clear, we have no reason to believe—and in fact, we do not believe—that this problem is more characteristic of the literature on the limited strength model than it might be of other literatures in psychology.

Importantly, taking advantage of researcher degrees of freedom seems to inflate funnel plot asymmetry: Bakker et al. (2012) simulated a literature in which the true effect was *d* = 0, and the production of “observed” experiments—that is, those that made it into the simulated meta-analyses—was influenced by publication bias. For these simulated data, the meta-analytic estimate of the true effect was *d* = 0.35, and the funnel plot was significantly asymmetric (the standardized asymmetry coefficient for Egger's regression test, which is analogous to *b*_1_ in PET, was 3.96, *p* < 0.001). When the use of researcher degrees of freedom was added to the simulation, the meta-analytic estimate was further inflated to *d* = 0.48, and the coefficient for Egger's regression increased to 6.07, *p* < 0.001. These results demonstrate that use of researcher degrees of freedom is, like publication bias, an example of a small-study effect (i.e., one way in which a correlation between effect sizes and standard errors might arise in a meta-analytic sample). Since the interaction of researcher degrees of freedom and publication bias creates an increase in funnel plot asymmetry, regression-based methods can be argued to produce an estimate of the underlying effect that is robust to both publication bias *and* the use of researcher degrees of freedom, whereas attempts at collecting unpublished data cannot account for the influence of the use of researcher degrees of freedom. To our knowledge, the use of regression-based methods to correct for the influence of the use of researcher degrees of freedom has yet to be formally assessed; however, it would seem to be a promising avenue for future work^3^.

We do not wish to imply that thorough attempts at collecting unpublished data are worthless (in fact, we are currently engaged in an effort to collect unpublished tests of the depletion effect in hopes of updating Hagger et al.'s conclusions in a manner that takes into account both published and unpublished results), but the ever-present specter of publication bias, as well as the apparently widespread use of researcher degrees of freedom (John et al., 2012), therefore means that statistical techniques such as the ones we employed here will continue to be essential to any endeavor to meta-analytically evaluate the evidence for any effect.

Based on responses from reviewers of previous drafts of this paper, as well as a commentary by Hagger and Chatzisarantis (2014) on our related work (Carter and McCullough, 2013b), we would like to anticipate and respond to some potential objections to our analyses and conclusions. First, some might argue that our claim that Hagger et al. (2010) have likely overestimated the magnitude of the depletion effect is unimportant because research in all areas of science is biased. Although we agree that bias is likely rampant, we hold that if it is worthwhile to conduct a meta-analysis on a topic, it is worthwhile to provide the most accurate estimate of the underlying effect as possible. We think there are good reasons to believe that the estimates we provide here are more accurate than those initially provided by Hagger et al. (2010).

Second, our argument that the depletion effect is indistinguishable from zero implies that a large number of experiments that have produced null or negative (i.e., performing self-control improves subsequent self-control) findings have been conducted but not reported. As mentioned, it is likely that some (perhaps many) null or negative results have been converted to positive findings via the use of researcher degrees of freedom, though Hagger and Chatzisarantis (2014) are skeptical that publication bias and the undisclosed exercise of researcher degrees of freedom could be as widespread in the ego depletion literature as what we are speculating here. However, given the difficulties inherent in determining the nature and number of unpublished findings (as discussed above), Hagger and Chatzisarantis's (2014) belief is based chiefly on their intuitions about how research in this area is conducted—specifically, that researchers, reviewers, and editors handling data or manuscripts on the limited strength model would view null or negative results as worth pursuing and publishing as is, rather than consigning such results to their hard drives or file drawers, or working to transform them into rejections of the null hypothesis through the exercise of researcher degrees of freedom. Since there is no empirical basis for Hagger and Chatzisarantis's argument [while there is an empirical basis for our argument that (1) the exercise of researcher degrees of freedom in psychology in general is widespread (John et al., 2012), and (2) many more rejections of the null hypothesis are appearing in at least some psychology journals than should be expected by chance (Francis, 2014)], their objection does not invalidate our conclusions, although it does highlight the importance of attempts at documenting the unpublished literature.

We do not wish to imply that we have unquestionably shown that the depletion effect is not a real phenomenon. The claim that the depletion effect is indistinguishable from zero is dependent on the validity of PET-PEESE, which, although promising, is still a relatively new method. Moreover, we are not suggesting that the limited strength model should be abandoned. Instead, we believe our results are best interpreted as demonstrating that the current evidence for the depletion effect is not convincing, despite the hundreds of experiments that have examined it.

We hope that the findings we present here will motivate researchers to re-examine the replicability and the magnitude of the depletion effect. Because our findings suggest that very large experiments will produce estimates of the depletion effect that are approximately zero, a useful next step would be a coordinated series of large, pre-registered direct replications of the original experiments (e.g., Baumeister et al., 1998). Pre-registering the methods for replications, as well as committing to making the data available regardless of their outcomes, would completely prevent publication bias. Given that our results support the conclusion that the depletion effect is approximately zero, it is difficult to know how big of a sample should be collected for these pre-registered replications or for any future experiments on the depletion effect. Regardless, researchers should be prepared to collect far larger samples than have been collected previously in this literature. For example, if we assume an overall effect of *d* = 0.25 (*b*_0_ from PEESE for the full sample), 80% power would be reached with *n* = 252 per condition. In contrast, the approximate average *n* per condition in Hagger et al. (2010)'s dataset was *n* = 27, with an approximate inter-quartile range spanning *n* = 17 to *n* = 31 per condition. In other words, if *b*_0_ from PEESE happened to be the correct estimate of the underlying effect size instead of the non-significant *b*_0_ from PET, 75% of the experiments in Hagger et al. (2010) would have needed to be *at least* 700% larger to obtain adequate power.

The broadest conclusion to be drawn from our findings is that unless methods for controlling publication bias and researcher degrees of freedom come to be taken more seriously, such as the development and use of statistical techniques (like PET-PEESE), some system for the required pre-registration of experiments—or until researchers, reviewers, and editors manage their aversion to the null hypothesis (Greenwald, 1975) through other measures—psychological science will likely falter in its efforts to develop trustworthy models, not only of self-control, but of every other psychological phenomenon as well.

### Conflict of interest statement

The authors declare that the research was conducted in the absence of any commercial or financial relationships that could be construed as a potential conflict of interest.

## Footnotes

^1^Hagger et al. (2010) modified three “outlier” effect sizes, which potentially obscured funnel plot asymmetry. Therefore, we used the original effect sizes. Using Hagger et al.'s (2010) transformed values leaves the results we report here essentially unchanged.

^2^Six of the 198 experiments could not be assigned to any of the four “sphere of self-control” categories. Therefore, following Hagger et al. (2010), we omitted these experiments from any subsample analyses (i.e., the subsample analyses are conducted on only 192 of the 198 experiments).

^3^A method based on the *p*-curve technique (Simonsohn et al., 2014a) is currently being developed for providing an estimate of the underlying effect that is robust to publication bias and the undisclosed use of researcher degrees of freedom. The *p*-curve method assumes that publication bias and the use of researcher degrees of freedom (also called “*p*-hacking”) result in *p*-values that are disproportionately close to the *p* < 0.05 criterion (e.g., more *p*-values within the interval of 0.04 < *p* < 0.05 than other intervals). This method is thoroughly discussed in a working paper by Simonsohn et al. (2014b). One point relevant to the depletion effect is that caution is required when using the *p*-curve technique on a literature that includes tests of attenuated interaction hypotheses (i.e., situations in which the effect of interest is thought to be reduced or eliminated at one level of a moderator, but not at another level; Simonsohn et al., 2014b). Many effect sizes in the Hagger et al. (2010) dataset were taken from the unattenuated simple effects of such interactions, and are ineligible for use with the *p*-curve method. These effects violate the core assumption of the *p*-curve analysis because publication bias and the use of researcher degrees of freedom are assumed to focus on making the *p*-value for the interaction effect just significant enough, which in turn requires the *p*-value for the unattenuated simple effect to be even smaller. Importantly, there is no explicit reason that these same effects would be inappropriately modeled by PET-PEESE, although it is not known whether their presence affects the accuracy of the estimates in any way.
