# Supplementary material for: Publication bias and the limited strength model of self-control: has the evidence for ego depletion been overestimated?
Source: Front Psychol. 2014 Jul 30;5:823. doi: 10.3389/fpsyg.2014.00823 (PMC4115664; doi:10.3389/fpsyg.2014.00823)
Supplement: Supplementary file 1 [file Presentation1.ZIP › Data Sheet.DOCX]

#Data were obtained from Martin Hagger, and further uses of #these data should be acknowledged as such.

#Citation: Hagger, M. S., Wood, C., Stiff, C., & Chatzisarantis, #N. L. D. (2010). Ego depletion and the strength model of self-#control: A meta-analysis. Psychological Bulletin, 136, 495–525.

#The necessary packages (please ensure that these have been #installed before running the following code).

library(pwr)

library(meta)

library(metafor)

#The data for the full sample. All outputs that end in ".all" #are based on the full sample.

d.all<-c(0.88,1.62,1.79,1.9,0.76,0.59,0.56,0.53,0.95,0.59,0.55,0.64,0.61,0.47,0.91,0.45,0.59,0.73,0.94,1.12,0.96,0.37,0.66,0.67,0.92,0.52,0.68,0.59,1.18,0.77,1.16,0.57,0.38,0.91,0.81,0.79,0.66,0.9,1.41,0.65,0.46,0.62,0.62,0.77,0.62,0.65,0.84,0.86,0.34,0.31,0.37,0.99,0.92,0.73,0.46,0.89,0.4,0.38,0.99,0.49,0.59,0.83,0.28,0.62,0.7,1.83,0.52,1.12,0.66,0.73,0.46,0.12,1.68,0.84,0.66,0.94,0.69,0.55,1,0.18,0.53,0.58,0.7,0.72,0.42,0.58,0.9,0.71,0.85,0.66,0.53,3.02,1.14,0.6,0.11,0.59,0.47,0.7,0.6,0.62,0.59,0.64,0.75,0.57,0.84,1.08,0.59,0.98,1.53,0.44,0.56,0.29,0.18,0.36,0.25,0.15,1.42,0.79,0.93,0.91,0.95,0.45,0.52,0.51,0.08,0.68,0.53,0.85,0.61,0.75,1.61,0.43,0.9,0.41,0.47,0.65,0.67,0.19,0.47,0.02,0.04,0.82,0.6,1.2,0.61,0.83,1.07,0.97,1.36,0.58,1.56,0.99,1.36,1.22,2.6,0.44,1.19,0.58,0.64,1.59,1.06,0.8,0.63,0.7,0.79,1.34,1.01,0.98,0.99,0.84,0.93,0.73,0.96,1.27,1.38,1.4,0.77,0.76,1.07,0.76,1.13,1.25,1.27,0.77,1.11,0.95,1.73,0.9,0.3,0.13,0.34,-0.11,0.21,0.21,-0.57,0.65,0.65,0.56)

n1.all<-c(20,20,22,10,15,42,25,34,14,22,45,22,21,36,11,39,30,19,12,14,15,73,17,27,26,49,20,30,23,19,54,50,23,13,27,23,16,15,8,50,49,28,26,15,25,28,18,24,35,24,16,14,11,31,37,20,49,127,10,34,29,10,10,29,28,12,24,16,21,33,36,26,18,21,31,54,41,20,19,50,30,34,19,17,33,42,39,13,28,21,29,8,24,15,80,23,17,19,22,21,12,30,17,26,18,36,37,19,20,45,32,142,251,53,32,81,9,30,16,17,15,38,31,29,29,15,32,25,30,36,12,19,18,37,28,42,28,51,19,20,17,30,31,23,24,15,17,15,15,30,30,20,10,10,10,10,20,34,29,15,30,17,29,36,16,15,15,13,13,20,20,24,18,35,20,9,14,18,24,24,12,13,14,25,21,16,14,34,23,23,26,24,18,19,26,26,33,40)

n2.all<-c(20,20,22,10,15,42,24,34,13,22,44,22,21,36,11,39,30,18,12,14,15,73,16,26,25,48,19,30,23,18,54,50,23,13,27,23,16,14,8,50,48,28,26,15,24,28,18,24,34,24,16,13,10,31,36,20,49,45,9,33,28,9,9,28,27,12,22,16,20,33,36,24,15,21,30,53,40,19,18,49,29,34,18,16,26,42,39,12,28,20,29,8,24,15,80,23,17,19,21,20,12,30,17,23,14,35,36,19,20,40,25,142,250,52,32,81,13,30,16,17,15,41,31,32,32,15,33,25,29,36,12,18,18,36,27,41,29,50,21,20,16,30,31,22,69,30,16,15,15,30,30,20,10,10,10,10,20,34,29,15,30,17,28,35,16,15,15,12,13,20,20,40,17,35,20,9,14,18,24,23,11,12,13,25,21,15,14,34,25,23,23,23,18,18,27,27,32,40)

d.v.all<-(((n1.all+n2.all)/(n1.all*n2.all))+(d.all^2/(2*(n1.all+n2.all))))

d.se.all<-sqrt(d.v.all)

#The data for the "controlling impulses" subsample. All outputs #that end in ".ci" are based on the controlling impulses #subsample.

d.ci<-c(0.88,1.62,1.79,1.9,0.59,0.56,0.53,0.95,0.59,0.55,0.64,0.61,0.47,0.73,0.94,0.66,0.67,0.92,1.18,0.77,0.66,1.41,0.34,0.31,0.37,0.92,0.73,0.59,0.52,1.12,0.12,1.68,0.84,0.66,0.53,0.7,0.72,0.9,0.71,0.53,0.6,0.11,0.59,0.7,0.6,0.62,0.59,0.64,0.75,1.08,0.59,0.44,1.42,0.79,0.93,0.91,0.95,0.61,0.75,0.41,0.47,0.65,0.67,0.19,0.02,0.04,0.82,0.6,1.2,0.61,0.83,1.07,0.97,1.56,1.36,1.22,2.6,0.44,1.19,0.64,1.06,1.34,1.01,0.98,0.99,0.84,1.27,1.38,1.4,0.77,0.76,1.07,0.76,1.13,1.25,1.27,0.77,1.11,0.95,1.73,0.3,0.34,-0.57,0.65)

n1.ci<-c(20,20,22,10,42,25,34,14,22,45,22,21,36,19,12,17,27,26,23,19,16,8,35,24,16,11,31,29,24,16,26,18,21,31,30,19,17,39,13,29,15,80,23,19,22,21,12,30,17,36,37,45,9,30,16,17,15,30,36,37,28,42,28,51,20,17,30,31,23,24,15,17,15,30,10,10,10,10,20,29,30,15,15,13,13,20,35,20,9,14,18,24,24,12,13,14,25,21,16,14,23,26,26,33)

n2.ci<-c(20,20,22,10,42,24,34,13,22,44,22,21,36,18,12,16,26,25,23,18,16,8,34,24,16,10,31,28,22,16,24,15,21,30,29,18,16,39,12,29,15,80,23,19,21,20,12,30,17,35,36,40,13,30,16,17,15,29,36,36,27,41,29,50,20,16,30,31,22,69,30,16,15,30,10,10,10,10,20,29,30,15,15,12,13,20,35,20,9,14,18,24,23,11,12,13,25,21,15,14,25,23,27,32)

d.v.ci<-(((n1.ci+n2.ci)/(n1.ci*n2.ci))+(d.ci^2/(2*(n1.ci+n2.ci))))

d.se.ci<-sqrt(d.v.ci)

#The data for the "cognitive processing" subsample. All outputs #that end in ".cp" are based on the cognitive processing #subsample.

d.cp<-c(0.76,0.59,0.59,0.81,0.79,0.9,0.62,0.65,0.84,0.86,0.46,0.89,0.4,0.38,0.83,0.28,0.62,0.7,0.66,0.46,0.69,0.55,1,0.58,0.84,1.53,0.45,0.52,0.51,0.08,0.53,0.85,1.61,0.43,0.9,0.47,0.99,0.58,0.93,0.73,0.9,0.13,-0.11,0.21,0.21,0.65,0.56)

n1.cp<-c(15,30,30,27,23,15,25,28,18,24,37,20,49,127,10,10,29,28,21,36,41,20,19,34,18,20,38,31,29,29,32,25,12,19,18,19,20,34,20,24,34,23,24,18,19,26,40)

n2.cp<-c(15,30,30,27,23,14,24,28,18,24,36,20,49,45,9,9,28,27,20,36,40,19,18,34,14,20,41,31,32,32,33,25,12,18,18,21,20,34,20,40,34,23,23,18,18,27,40)

d.v.cp<-(((n1.cp+n2.cp)/(n1.cp*n2.cp))+(d.cp^2/(2*(n1.cp+n2.cp))))

d.se.cp<-sqrt(d.v.cp)

#The data for the "choice and volition" subsample. All outputs #that end in ".cv" are based on the choice and volition #subsample.

d.cv<-c(0.91,0.18,0.42,0.29,0.18,0.36,0.25,0.15)

n1.cv<-c(13,50,33,142,251,53,32,81)

n2.cv<-c(13,49,26,142,250,52,32,81)

d.v.cv<-(((n1.cv+n2.cv)/(n1.cv*n2.cv))+(d.cv^2/(2*(n1.cv+n2.cv))))

d.se.cv<-sqrt(d.v.cv)

#The data for the "social processing" subsample. All outputs #that end in ".sp" are based on the social processing subsample.

d.sp<-c(0.91,0.45,1.12,0.96,0.37,0.52,0.68,1.16,0.57,0.38,0.65,0.46,0.62,0.62,0.77,0.99,0.99,0.49,1.83,0.73,0.94,0.58,0.85,0.66,0.98,0.56,1.36,0.58,0.8,0.63,0.7,0.79,0.96)

n1.sp<-c(11,39,14,15,73,49,20,54,50,23,50,49,28,26,15,14,10,34,12,33,54,42,28,21,19,32,15,30,17,29,36,16,18)

n2.sp<-c(11,39,14,15,73,48,19,54,50,23,50,48,28,26,15,13,9,33,12,33,53,42,28,20,19,25,15,30,17,28,35,16,17)

d.v.sp<-(((n1.sp+n2.sp)/(n1.sp*n2.sp))+(d.sp^2/(2*(n1.sp+n2.sp))))

d.se.sp<-sqrt(d.v.sp)

#Fixed-effect and Random-effects meta-analysis

meta.all<-metagen(d.all, d.se.all)

meta.ci<-metagen(d.ci, d.se.ci)

meta.cp<-metagen(d.cp, d.se.cp)

meta.cv<-metagen(d.cv, d.se.cv)

meta.sp<-metagen(d.sp, d.se.sp)

#Results from FE and RE models

meta.all

meta.ci

meta.cp

meta.cv

meta.sp

#Trim and fill

tf.all<-trimfill(meta.all)

tf.ci<-trimfill(meta.ci)

tf.cp<-trimfill(meta.cp)

tf.cv<-trimfill(meta.cv)

tf.sp<-trimfill(meta.sp)

#Results from trim and fill

tf.all

tf.ci

tf.cp

tf.cv

tf.sp

#Deriving individual CIs for each experiment and the number of #studies where p>.05

ci.all<-data.frame(ci(TE = d.all, seTE = d.se.all))

ci.ci<-data.frame(ci(TE = d.ci, seTE = d.se.ci))

ci.cp<-data.frame(ci(TE = d.cp, seTE = d.se.cp))

ci.cv<-data.frame(ci(TE = d.cv, seTE = d.se.cv))

ci.sp<-data.frame(ci(TE = d.sp, seTE = d.se.sp))

NumNonSig.all<-with(ci.all, c(sum(p>.05)))

NumSig.all<-198-NumNonSig.all

NumSig.all

NumNonSig.ci<-with(ci.ci, c(sum(p>.05)))

NumSig.ci<-104-NumNonSig.ci

NumSig.ci

NumNonSig.cp<-with(ci.cp, c(sum(p>.05)))

NumSig.cp<-47-NumNonSig.cp

NumSig.cp

NumNonSig.cv<-with(ci.cv, c(sum(p>.05)))

NumSig.cv<-8-NumNonSig.cv

NumSig.cv

NumNonSig.sp<-with(ci.sp, c(sum(p>.05)))

NumSig.sp<-33-NumNonSig.sp

NumSig.sp

#Calculating average power based on effect size estimates for #individual experiments

P.ind.all<-mapply(pwr.t2n.test, d=d.all, n1 = n1.all, n2 = n2.all)

PowP.ind.all<-sapply(P.ind.all[5,1:198], as.numeric)

MeanPowerInd.all<-mean(PowP.ind.all)

MeanPowerInd.all

#count of powers>.8

length(which(PowP.ind.all>=.8))

P.ind.ci<-mapply(pwr.t2n.test, d=d.ci, n1 = n1.ci, n2 = n2.ci)

PowP.ind.ci<-sapply(P.ind.ci[5,1:104], as.numeric)

MeanPowerInd.ci<-mean(PowP.ind.ci)

MeanPowerInd.ci

#count of powers>.8

length(which(PowP.ind.ci>=.8))

P.ind.cp<-mapply(pwr.t2n.test, d=d.cp, n1 = n1.cp, n2 = n2.cp)

PowP.ind.cp<-sapply(P.ind.cp[5,1:47], as.numeric)

MeanPowerInd.cp<-mean(PowP.ind.cp)

MeanPowerInd.cp

#count of powers>.8

length(which(PowP.ind.cp>=.8))

P.ind.cv<-mapply(pwr.t2n.test, d=d.cv, n1 = n1.cv, n2 = n2.cv)

PowP.ind.cv<-sapply(P.ind.cv[5,1:8], as.numeric)

MeanPowerInd.cv<-mean(PowP.ind.cv)

MeanPowerInd.cv

#count of powers>.8

length(which(PowP.ind.cv>=.8))

P.ind.sp<-mapply(pwr.t2n.test, d=d.sp, n1 = n1.sp, n2 = n2.sp)

PowP.ind.sp<-sapply(P.ind.sp[5,1:33], as.numeric)

MeanPowerInd.sp<-mean(PowP.ind.sp)

MeanPowerInd.sp

#count of powers>.8

length(which(PowP.ind.sp>=.8))

#Calculating average power based on post-hoc power calculations #assuming FE estimate of d = .62 for full sample

P.62.all<-mapply(pwr.t2n.test, d=.62, n1 = n1.all, n2 = n2.all)

PowP.62.all<-sapply(P.62.all[5,1:198], as.numeric)

MeanPower.62.all<-mean(PowP.62.all)

MeanPower.62.all

#count of powers>.8

length(which(PowP.62.all>=.8))

#Calculating average power based on post-hoc power calculations #assuming RE estimate of d = .68 for full sample

P.68.all<-mapply(pwr.t2n.test, d=.68, n1 = n1.all, n2 = n2.all)

PowP.68.all<-sapply(P.68.all[5,1:198], as.numeric)

MeanPower.68.all<-mean(PowP.68.all)

MeanPower.68.all

#count of powers>.8

length(which(PowP.68.all>=.8))

#Calculating average power based on post-hoc power calculations #assuming FE estimate of d = .71 for controlling impulses #subsample

P.71.ci<-mapply(pwr.t2n.test, d=.71, n1 = n1.ci, n2 = n2.ci)

PowP.71.ci<-sapply(P.71.ci[5,1:104], as.numeric)

MeanPower.71.ci<-mean(PowP.71.ci)

MeanPower.71.ci

#count of powers>.8

length(which(PowP.71.ci>=.8))

#Calculating average power based on post-hoc power calculations #assuming RE estimate of d = .75 for controlling impulses #subsample

P.75.ci<-mapply(pwr.t2n.test, d=.75, n1 = n1.ci, n2 = n2.ci)

PowP.75.ci<-sapply(P.75.ci[5,1:104], as.numeric)

MeanPower.75.ci<-mean(PowP.75.ci)

MeanPower.75.ci

#count of powers>.8

length(which(PowP.75.ci>=.8))

#Calculating average power based on post-hoc power calculations #assuming FE estimate of d = .6 for cognitive processing #subsample

P.6.cp<-mapply(pwr.t2n.test, d=.6, n1 = n1.cp, n2 = n2.cp)

PowP.6.cp<-sapply(P.6.cp[5,1:47], as.numeric)

MeanPower.6.cp<-mean(PowP.6.cp)

MeanPower.6.cp

#count of powers>.8

length(which(PowP.6.cp>=.8))

#Calculating average power based on post-hoc power calculations #assuming FE estimate of d = .24 for choice and volition #subsample

P.24.cv<-mapply(pwr.t2n.test, d=.24, n1 = n1.cv, n2 = n2.cv)

PowP.24.cv<-sapply(P.24.cv[5,1:8], as.numeric)

MeanPower.24.cv<-mean(PowP.24.cv)

MeanPower.24.cv

#count of powers>.8

length(which(PowP.24.cv>=.8))

#Calculating average power based on post-hoc power calculations #assuming FE estimate of d = .69 for social processing subsample

P.69.sp<-mapply(pwr.t2n.test, d=.69, n1 = n1.sp, n2 = n2.sp)

PowP.69.sp<-sapply(P.69.sp[5,1:33], as.numeric)

MeanPower.69.sp<-mean(PowP.69.sp)

MeanPower.69.sp

#count of powers>.8

length(which(PowP.69.sp>=.8))

#p values for binomial tests

Powers.all<-c(MeanPowerInd.all, MeanPower.62.all, MeanPower.68.all)

Powers.all

BinomPowInd.all<-mapply(binom.test, NumSig.all, 198, Powers.all, conf.level = .9, alternative = "greater")

p.values.all<-sapply(BinomPowInd.all[3,1:3], as.numeric)

p.values.all

Powers.ci<-c(MeanPowerInd.ci, MeanPower.71.ci, MeanPower.75.ci)

Powers.ci

BinomPowInd.ci<-mapply(binom.test, NumSig.ci, 104, Powers.ci, conf.level = .9, alternative = "greater")

p.values.ci<-sapply(BinomPowInd.ci[3,1:3], as.numeric)

p.values.ci

Powers.cp<-c(MeanPowerInd.cp, MeanPower.6.cp)

Powers.cp

BinomPowInd.cp<-mapply(binom.test, NumSig.cp, 47, Powers.cp, conf.level = .9, alternative = "greater")

p.values.cp<-sapply(BinomPowInd.cp[3,1:2], as.numeric)

p.values.cp

Powers.cv<-c(MeanPowerInd.cv, MeanPower.24.cv)

Powers.cv

BinomPowInd.cv<-mapply(binom.test, NumSig.cv, 8, Powers.cv, conf.level = .9, alternative = "greater")

p.values.cv<-sapply(BinomPowInd.cv[3,1:2], as.numeric)

p.values.cv

Powers.sp<-c(MeanPowerInd.sp, MeanPower.69.sp)

Powers.sp

BinomPowInd.sp<-mapply(binom.test, NumSig.sp, 33, Powers.sp, conf.level = .9, alternative = "greater")

p.values.sp<-sapply(BinomPowInd.sp[3,1:2], as.numeric)

p.values.sp

#PET

SE.all<-lm(d.all~d.se.all, weights = 1/d.v.all)

summary(SE.all)

confint(SE.all)

SE.ci<-lm(d.ci~d.se.ci, weights = 1/d.v.ci)

summary(SE.ci)

confint(SE.ci)

SE.cp<-lm(d.cp~d.se.cp, weights = 1/d.v.cp)

summary(SE.cp)

confint(SE.cp)

SE.cv<-lm(d.cv~d.se.cv, weights = 1/d.v.cv)

summary(SE.cv)

confint(SE.cv)

SE.sp<-lm(d.sp~d.se.sp, weights = 1/d.v.sp)

summary(SE.sp)

confint(SE.sp)

#PEESE

v.all<-lm(d.all~d.v.all, weights = 1/d.v.all)

summary(v.all)

confint(v.all)

v.ci<-lm(d.ci~d.v.ci, weights = 1/d.v.ci)

summary(v.ci)

confint(v.ci)

v.cp<-lm(d.cp~d.v.cp, weights = 1/d.v.cp)

summary(v.cp)

confint(v.cp)

v.cv<-lm(d.cv~d.v.cv, weights = 1/d.v.cv)

summary(v.cv)

confint(v.cv)

v.sp<-lm(d.sp~d.v.sp, weights = 1/d.v.sp)

summary(v.sp)

confint(v.sp)

#Funnel plot

#This will save as a .png file in the documents folder

png(file="funnel.png", width = 1600, height = 1400, res = 200)

layout(matrix(c(1,1,2,3,4,5), 3, 2, byrow = TRUE), respect = TRUE)

funnel(meta.all, lty.fixed = 1, lty.random = 5, xlim = c(-3.1, 3.1), xlab = "Effect size", ylab = "Standard Error (SE)", cex = .75, col = 1, bg = 1, contour=c(0.00001, 0.95), col.contour=c("grey", "white"), family = "serif", cex.lab = 1.5, main = "Full Sample", cex.main = 1.8)

funnel(meta.ci, lty.fixed = 1, lty.random = 5, xlim = c(-3.1, 3.1), xlab = "Effect size", ylab = "Standard Error (SE)", cex = .75, col = 1, bg = 1, contour=c(0.00001, 0.95), col.contour=c("grey", "white"), family = "serif", cex.lab = 1.5, main = "Controlling Impulses", cex.main = 1.8)

funnel(meta.cp, lty.fixed = 1, lty.random = 5, xlim = c(-2, 2), xlab = "Effect size", ylab = "Standard Error (SE)", cex = .75, col = 1, bg = 1, contour=c(0.00001, 0.95), col.contour=c("grey", "white"), family = "serif", cex.lab = 1.5, main = "Cognitive Processing", cex.main = 1.8)

funnel(meta.cv, lty.fixed = 1, lty.random = 5, xlim = c(-1, 1), xlab = "Effect size", ylab = "Standard Error (SE)", cex = .75, col = 1, bg = 1, contour=c(0.00001, 0.95), col.contour=c("grey", "white"), family = "serif", cex.lab = 1.5, main = "Choice and Volition", cex.main = 1.8)

funnel(meta.sp, lty.fixed = 1, lty.random = 5, xlim = c(-2, 2), xlab = "Effect size", ylab = "Standard Error (SE)", cex = .75, col = 1, bg = 1, contour=c(0.00001, 0.95), col.contour=c("grey", "white"), family = "serif", cex.lab = 1.5, main = "Social Processing", cex.main = 1.8)

dev.off()

#Data used by Hagger et al. (2010) with outliers transformed. #Outliers were present only in the full sample and the #controlling impulses subsample.

#Simply use the following lines to specify the data before #running the above code.

#The data for the full sample without the outliers corrected. #All outputs that end in ".all" are based on the full sample.

d.all<-c(0.88,1.62,1.79,1.9,0.76,0.59,0.56,0.53,0.95,0.59,0.55,0.64,0.61,0.47,0.91,0.45,0.59,0.73,0.94,1.12,0.96,0.37,0.66,0.67,0.92,0.52,0.68,0.59,1.18,0.77,1.16,0.57,0.38,0.91,0.81,0.79,0.66,0.9,1.41,0.65,0.46,0.62,0.62,0.77,0.62,0.65,0.84,0.86,0.34,0.31,0.37,0.99,0.92,0.73,0.46,0.89,0.4,0.38,0.99,0.49,0.59,0.83,0.28,0.62,0.7,1.83,0.52,1.12,0.66,0.73,0.46,0.12,1.68,0.84,0.66,0.94,0.69,0.55,1,0.18,0.53,0.58,0.7,0.72,0.42,0.58,0.9,0.71,0.85,0.66,0.53,1.9,1.14,0.6,0.11,0.59,0.47,0.7,0.6,0.62,0.59,0.64,0.75,0.57,0.84,1.08,0.59,0.98,1.53,0.44,0.56,0.29,0.18,0.36,0.25,0.15,1.42,0.79,0.93,0.91,0.95,0.45,0.52,0.51,0.08,0.68,0.53,0.85,0.61,0.75,1.61,0.43,0.9,0.41,0.47,0.65,0.67,0.19,0.47,0.02,0.04,0.82,0.6,1.2,0.61,0.83,1.07,0.97,1.36,0.58,1.56,0.99,1.36,1.22,1.9,0.44,1.19,0.58,0.64,1.59,1.06,0.8,0.63,0.7,0.79,1.34,1.01,0.98,0.99,0.84,0.93,0.73,0.96,1.27,1.38,1.4,0.77,0.76,1.07,0.76,1.13,1.25,1.27,0.77,1.11,0.95,1.73,0.9,0.3,0.13,0.34,-0.11,0.21,0.21,-.11,0.65,0.65,0.56)

n1.all<-c(20,20,22,10,15,42,25,34,14,22,45,22,21,36,11,39,30,19,12,14,15,73,17,27,26,49,20,30,23,19,54,50,23,13,27,23,16,15,8,50,49,28,26,15,25,28,18,24,35,24,16,14,11,31,37,20,49,127,10,34,29,10,10,29,28,12,24,16,21,33,36,26,18,21,31,54,41,20,19,50,30,34,19,17,33,42,39,13,28,21,29,8,24,15,80,23,17,19,22,21,12,30,17,26,18,36,37,19,20,45,32,142,251,53,32,81,9,30,16,17,15,38,31,29,29,15,32,25,30,36,12,19,18,37,28,42,28,51,19,20,17,30,31,23,24,15,17,15,15,30,30,20,10,10,10,10,20,34,29,15,30,17,29,36,16,15,15,13,13,20,20,24,18,35,20,9,14,18,24,24,12,13,14,25,21,16,14,34,23,23,26,24,18,19,26,26,33,40)

n2.all<-c(20,20,22,10,15,42,24,34,13,22,44,22,21,36,11,39,30,18,12,14,15,73,16,26,25,48,19,30,23,18,54,50,23,13,27,23,16,14,8,50,48,28,26,15,24,28,18,24,34,24,16,13,10,31,36,20,49,45,9,33,28,9,9,28,27,12,22,16,20,33,36,24,15,21,30,53,40,19,18,49,29,34,18,16,26,42,39,12,28,20,29,8,24,15,80,23,17,19,21,20,12,30,17,23,14,35,36,19,20,40,25,142,250,52,32,81,13,30,16,17,15,41,31,32,32,15,33,25,29,36,12,18,18,36,27,41,29,50,21,20,16,30,31,22,69,30,16,15,15,30,30,20,10,10,10,10,20,34,29,15,30,17,28,35,16,15,15,12,13,20,20,40,17,35,20,9,14,18,24,23,11,12,13,25,21,15,14,34,25,23,23,23,18,18,27,27,32,40)

d.v.all<-(((n1.all+n2.all)/(n1.all*n2.all))+(d.all^2/(2*(n1.all+n2.all))))

d.se.all<-sqrt(d.v.all)

#The data for the "controlling impulses" subsample without the #outliers corrected. All outputs that end in ".ci" are based on #the controlling impulses subsample.

d.ci<-c(0.88,1.62,1.79,1.9,0.59,0.56,0.53,0.95,0.59,0.55,0.64,0.61,0.47,0.73,0.94,0.66,0.67,0.92,1.18,0.77,0.66,1.41,0.34,0.31,0.37,0.92,0.73,0.59,0.52,1.12,0.12,1.68,0.84,0.66,0.53,0.7,0.72,0.9,0.71,0.53,0.6,0.11,0.59,0.7,0.6,0.62,0.59,0.64,0.75,1.08,0.59,0.44,1.42,0.79,0.93,0.91,0.95,0.61,0.75,0.41,0.47,0.65,0.67,0.19,0.02,0.04,0.82,0.6,1.2,0.61,0.83,1.07,0.97,1.56,1.36,1.22,1.9,0.44,1.19,0.64,1.06,1.34,1.01,0.98,0.99,0.84,1.27,1.38,1.4,0.77,0.76,1.07,0.76,1.13,1.25,1.27,0.77,1.11,0.95,1.73,0.3,0.34,-0.11,0.65)

n1.ci<-c(20,20,22,10,42,25,34,14,22,45,22,21,36,19,12,17,27,26,23,19,16,8,35,24,16,11,31,29,24,16,26,18,21,31,30,19,17,39,13,29,15,80,23,19,22,21,12,30,17,36,37,45,9,30,16,17,15,30,36,37,28,42,28,51,20,17,30,31,23,24,15,17,15,30,10,10,10,10,20,29,30,15,15,13,13,20,35,20,9,14,18,24,24,12,13,14,25,21,16,14,23,26,26,33)

n2.ci<-c(20,20,22,10,42,24,34,13,22,44,22,21,36,18,12,16,26,25,23,18,16,8,34,24,16,10,31,28,22,16,24,15,21,30,29,18,16,39,12,29,15,80,23,19,21,20,12,30,17,35,36,40,13,30,16,17,15,29,36,36,27,41,29,50,20,16,30,31,22,69,30,16,15,30,10,10,10,10,20,29,30,15,15,12,13,20,35,20,9,14,18,24,23,11,12,13,25,21,15,14,25,23,27,32)

d.v.ci<-(((n1.ci+n2.ci)/(n1.ci*n2.ci))+(d.ci^2/(2*(n1.ci+n2.ci))))

d.se.ci<-sqrt(d.v.ci)
