# Supplementary material for: Publication bias and the limited strength model of self-control: has the evidence for ego depletion been overestimated?
Source: Front Psychol. 2014 Jul 30;5:823. doi: 10.3389/fpsyg.2014.00823 (PMC4115664; doi:10.3389/fpsyg.2014.00823)
Supplement: Supplementary file 1 [file Presentation1.ZIP › Appendix.PDF]

## 1. Appendix

PET and PEESE are regression models that are applied to meta-analytic datasets—that is, datasets for which each entry includes both an independent estimate of the effect size of interest (e.g., Cohen's *d*) and the standard error (*SE*) of that estimate.

If run as a WLS model, effect size (e.g., *d*) is predicted by its corresponding *SE*, for PET, or variance ($SE^2$), for PEESE, and the regression weights are set to the inverse of the variance ($1/SE^2$).

Formally, PET can be written as

$$d_i = b_0 + b_1 SE_i + \epsilon_i \,,$$

and PEESE as

$$d_i = b_0 + b_1 SE^2{}_i + \epsilon_i \,.$$

In both of the above models, the intercept, $b_0$, provides an estimate of the true underlying effect that is corrected for the influence of small-study effects. Additionally, in PET, the slope coefficient, $b_1$, functions as a test for the existence of small-study effects or funnel plot asymmetry (use of the slope coefficient in this way has been called Egger's regression test [Egger et al., 1997]).

If a researcher does not have access to software for running WLS regression, or for some reason prefers the more standard approach, both PET and PEESE can be run as ordinary least squares (OLS) models. For PET, the ratio of effect size to standard error (notated as *t*) is predicted by the inverse of standard error (1/*SE*), or

$$t_i = b_0 + b_1 \frac{1}{SE_i} + \epsilon_i \,.$$

In contrast to the WLS version of PET, here the intercept, $b_0$, is interpreted as a test for funnel plot asymmetry and the slope coefficient, $b_1$, as the corrected estimate of the true underlying effect. For PEESE, *t* is modelled as

$$t_i = b_0 + b_1 SE_i + b_2 \frac{1}{SE} + \epsilon_i \,,$$

where $b_0$ is constrained to zero and $b_2$ (the slope coefficient for the inverse of standard error) is the corrected estimate of the true underlying effect.

All of these models can be run in the free statistical language R. For example, given a list (i.e., vector) of effect sizes, `d`, a list of the associated standard errors, `se`, and a list of the associated variances, `v`, the WLS form of PET can be run as a

```
PET = lm(d~se, weights = 1/v)
```

and the WLS form of PEESE can be run as

```
PEESE = lm(d~v, weights = 1/v).
```

The results of the above code are two linear model objects titled “PET” and “PEESE.” Both objects can be run through functions such as `summary()` or `confint()` to produce a summary of the model results or confidence intervals around the intercept and slope coefficients, respectively. For example,

```
Summary(PET).
```

The models in the main text were run in this way, and we refer readers to the Data Sheet document in the supplemental materials for full examples of using PET and PEESE in this form.
